# Metabolically Engineered *Escherichia coli* for Conversion of D-Fructose to D-Allulose *via* Phosphorylation-Dephosphorylation

**DOI:** 10.3389/fbioe.2022.947469

**Published:** 2022-06-22

**Authors:** Qiang Guo, Chen-Yang Liu, Ling-Jie Zheng, Shang-He Zheng, Ya-Xing Zhang, Su-Ying Zhao, Hui-Dong Zheng, Li-Hai Fan, Xiao-Cheng Lin

**Affiliations:** ^1^ Fujian Engineering Research Center of Advanced Manufacturing Technology for Fine Chemicals, College of Chemical Engineering, Fuzhou University, Fuzhou, China; ^2^ Qingyuan Innovation Laboratory, Quanzhou, China

**Keywords:** D-allulose, *Escherichia coli*, fermentation, metabolic engineering, cell factory

## Abstract

D-Allulose is an ultra-low calorie sweetener with broad market prospects. As an alternative to Izumoring, phosphorylation-dephosphorylation is a promising method for D-allulose synthesis due to its high conversion of substrate, which has been preliminarily attempted in enzymatic systems. However, *in vitro* phosphorylation-dephosphorylation requires polyphosphate as a phosphate donor and cannot completely deplete the substrate, which may limit its application in industry. Here, we designed and constructed a metabolic pathway in *Escherichia coli* for producing D-allulose from D-fructose via *in vivo* phosphorylation-dephosphorylation. PtsG-F and Mak were used to replace the fructose phosphotransferase systems (PTS) for uptake and phosphorylation of D-fructose to fructose-6-phosphate, which was then converted to D-allulose by AlsE and A6PP. The D-allulose titer reached 0.35 g/L and the yield was 0.16 g/g. Further block of the carbon flux into the Embden-Meyerhof-Parnas (EMP) pathway and introduction of an ATP regeneration system obviously improved fermentation performance, increasing the titer and yield of D-allulose to 1.23 g/L and 0.68 g/g, respectively. The *E. coli* cell factory cultured in M9 medium with glycerol as a carbon source achieved a D-allulose titer of ≈1.59 g/L and a yield of ≈0.72 g/g on D-fructose.

## Introduction

D-Allulose is a United States Food and Drug Administration (FDA)-approved sweetener with ultra-low calorie content and unique physiological functions (Sun et al., 2005; Mu et al., 2012; Nagata et al., 2015), thus considered as a potential alternative to sucrose and owning broad market prospects (Su et al., 2018). Currently, D-allulose is industrially produced from D-fructose through enzymatic Izumoring (Wang et al., 2020), which reversibly epimerizes D-fructose at its C-3 position using D-allulose 3-epimerase (DAE) or D-tagatose 3-epimerase (DTE) as a biocatalyst (Mu et al., 2011). The major drawback of Izumoring is the low conversion of D-fructose, only reaching around 30% at equilibrium (Kim et al., 2006; Bosshart et al., 2012; Zhang et al., 2013). Continuous removal of D-allulose from the reaction system may improve conversion efficiency, but the coupling of isomer separation with Izumoring epimerization will definitely increase product cost (Nguyen et al., 2009; Li et al., 2018).

Recently, an *in vitro* enzymatic biosystem based on phosphorylation-dephosphorylation has been proposed for producing D-allulose from starch (Li et al., 2021). This strategy differed from Izumoring in its innovative use of epimerization between hexose monophosphates. After obtaining fructose-6-phosphate through phosphorylation and other reactions, allulose-6-phosphate epimerase (A6PE) catalyzed the reversible conversion of fructose-6-phosphate to allulose-6-phosphate, which was then dephosphorylated by allulose-6-phosphate phosphatase (A6PP) to generate D-allulose (Li et al., 2021). It is quite important that dephosphorylation was irreversible (You et al., 2017), which broke the epimerization equilibrium and drove the reactions towards target product. Although the resulting conversion ratio of D-fructose was much higher than with Izumoring, a certain amount of D-fructose would remain in products (<18%) since A6PP also exhibited a side activity on dephosphorylating fructose-6-phosphate (Li et al., 2021). Moreover, the enzymatic phosphorylation reaction in this system required expensive polyphosphate (polyP) as a phosphate donor (Shiba et al., 2000; Xiao et al., 2019), which might limit its large-scale application.

In contrast to enzymatic approach, fermentation may be a way that can fully release the application potential of phosphorylation-dephosphorylation strategy in the production of D-allulose from D-fructose, in which rational design of a microbial cell factory is the key to ensuring high fermentation performance (Lee et al., 2019; Xu et al., 2020). *Escherichia coli* is one of the most widely used bacterial hosts for cell factory development due to its clear genetic background, easy manipulation, and rapid growth (Pontrelli et al., 2018). *E. coli* can utilize D-fructose as a sole carbon source and has three routes for D-fructose uptake and phosphorylation (Aristidou et al., 1999; Kornberg, 2001), two of which are phosphoenolpyruvate (PEP): carbohydrate phosphotransferase systems (PTS). The fructose PTS (*fruA*, *fruB*) transports and concomitantly phosphorylates D-fructose to fructose-1-phosphate (Luo et al., 2014). The mannose PTS (*manXYZ*) can recognize sugars with the 3, 4, 5-D-arabino-hexose configuration (Kornberg, 2001), and the D-fructose taken up by this system appears in cells as fructose 6-phosphate (Kornberg, 1990). The fructose PTS and mannose PTS use PEP as a phosphate donor, and the resulting fructose monophosphates will be further phosphorylated to fructose-1, 6-bisphosphate before entering the central metabolic pathway (Luo et al., 2014). In addition to PTS, a mutant of glucose permease involved in the glucose PTS, which is specified by *ptsG*-*F*, has been found to possess the ability to transport D-fructose via facilitated diffusion (Kornberg et al., 2000). Once D-fructose is passaged, it can be phosphorylated to fructose 6-phosphate by an intracellular fructo/manno kinase (*mak*) with ATP as a phosphate donor (Miller and Raines, 2004). Therefore, the well-defined mechanisms for D-fructose transport and phosphorylation provide a prerequisite for application of phosphorylation-dephosphorylation in *E. coli*. Also, this bacterium is reported to own an endogenous allulose-6-phosphate epimerase encoded by *alsE*, whose kinetic parameters, substrate specificity and metal ion preference have been comprehensively investigated (Chan et al., 2008), suggesting that it is possible for wild-type *E. coli* to efficiently convert fructose-6-phosphate to allulose-6-phosphate.

**FIGURE 1:**
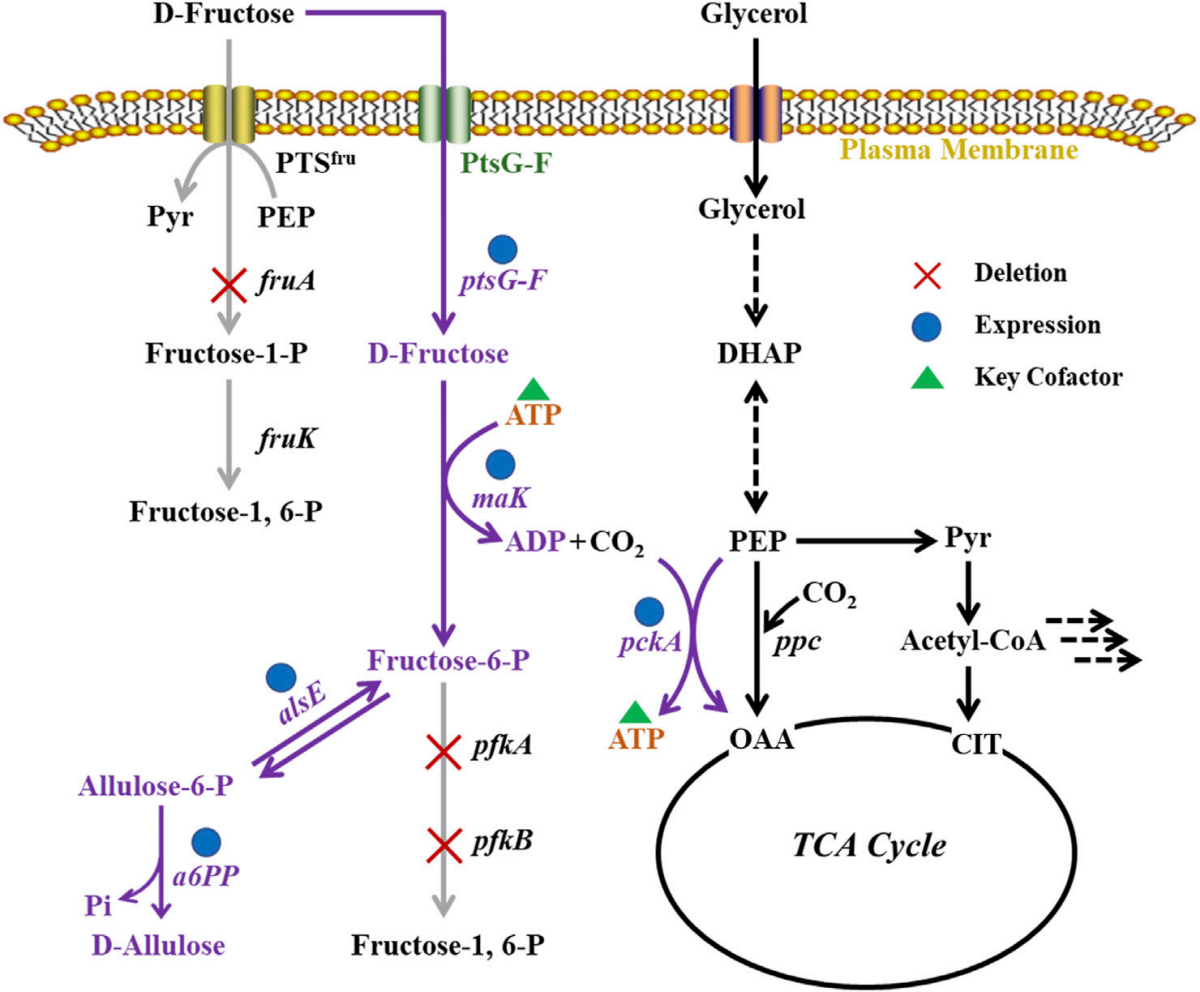
Metabolic pathways in *E. coli* cell factory for production of D-allulose from D-fructose *via* phosphorylation-dephosphorylation.

In this work, we designed a metabolic pathway for D-allulose production from D-fructose via phosphorylation-dephosphorylation in *E. coli* JM109 (DE3) (Figure 1). The fructose PTS was substituted by use of *ptsG*-*F* and *mak*, ensuring that D-fructose could be taken up and phosphorylated to fructose-6-phosphate by cells. The *E. coli alsE* was employed to catalyze the reversible reaction from fructose-6-phosphate to allulose-6-phosphate, followed by dephosphorylation by an allulose-6-phosphate phosphatase from *Bacteroides fragilis* (Li et al., 2021). The by-product synthesis pathways and the Embden-Meyerhof-Parnas (EMP) pathway were blocked to increase the yield of product. A PEP carboxykinase (*pckA*) from *Actinobacillus succinogenes* (Singh et al., 2011), which catalyzed the formation of oxaloacetate (OAA) and ATP from PEP, CO_2,_ and ADP, was introduced in *E. coli* cell factory to build an ATP regeneration system, with the purpose to enhance the supply of ATP as a phosphate donor for D-fructose phosphorylation. This work is expected to provide a strategy for efficient fermentative production of D-allulose, and the research achievements will promote the application of metabolic engineering in green biomanufacturing.

## Materials and Methods

### Strains and Media

The strains used in this study were listed in Table 1. *E. coli* Trans 10 was used for plasmid construction. *E. coli* JM109 (DE3) was employed as a host for protein expression and D-allulose production. Luria-Bertani (LB) medium was composed of 10.00 g/L NaCl, 10.00 g/L tryptone, and 5.00 g/L yeast extract. M9 minimal medium contained 0.50 g/L NaCl, 0.50 g/L NH_4_Cl, 0.84 mg/L ZnCl_2_, 44.10 mg/L CaCl_2_ 2H_2_O, 8.30 mg/L FeCl_3_·6H_2_O, 0.13 mg/L CuCl_2_·2H_2_O, 0.10 mg/L CoCl_2_·2H_2_O, 0.02 mg/L MnCl_2_·4H_2_O, 0.25 g/L MgSO_4_·7H_2_O, 0.10 mg/L H_3_BO_3_, 7.52 g/L Na_2_HPO_4_·2H_2_O, 3.00 g/L KH_2_PO_4_, 50.00 mg/L EDTA, 1.00 mg/L thiamin, and 1.00 mg/L biotin.

**TABLE 1:** The strains and plasmids used in this study.

Name	Relevant characteristics	References
Strains		
*E. coli* Trans 10	F^−^ *mcr*A Δ(*mrr*-*hsd*RMS-*mcr*BC) φ80 *lac*ZΔM15 Δ*lac*X74 *rec*A1 *ara*Δ139 Δ(*ara*-*leu*)7697 *gal*U *gal*K *rps*L (Str^R^) *end*A1 *nup*G	Trans gen biotech
*E. coli* JM109 (DE3)	*EndA1 recA1 gyr*A96 *thi hsdR17* (r_k_^−^, m_k_^+^) *relA1 sup*E44 λ^−^ Δ(*lac*-*pro*AB) [F′ *tra*D36 *proAB lac*I^q^ Δ(*lac*Z)M15] λ(DE3)	BeNa culture collection
*E. coli* (*control*)	*E. coli* JM109 (DE3) harboring pRSFDuet-1	This study
*E. coli* (*a6PP*)	*E. coli* JM109 (DE3) harboring pRSFDuet-*a6PP*	This study
*E. coli* (*alsE*)	*E. coli* JM109 (DE3) harboring pRSFDuet-*alsE*	This study
*E. coli* (*alsE*, *a6PP*)	*E. coli* JM109 (DE3) harboring pRSFDuet-*alsE*-*a6PP*	This study
*E. coli* (*alsE*, *a6PP*, Δ*fruA*)	*E. coli* JM109 (DE3) harboring pETDuet-1 and pRSFDuet-*alsE*-*a6PP* with deletion of *fruA*	This study
*E. coli* (*alsE*, *a6PP*, *ptsG*-*F*, *mak*, Δ*fruA*)	*E. coli* JM109 (DE3) harboring pETDuet-*ptsG*-*F*-*mak* and pRSFDuet-*alsE*-*a6PP* with deletion of *fruA*	This study
*E. coli* (*alsE*, *a6PP*, *ptsG*-*F*, *mak*, Δ*fruA*, Δ*pfkA*)	*E. coli* JM109 (DE3) harboring pETDuet-*ptsG*-*F*-*mak* and pRSFDuet-*alsE*-*a6PP* with deletion of *fruA* and *pfkA*	This study
*E. coli* (*alsE*, *a6PP*, *ptsG*-*F*, *mak*, Δ*fruA*, Δ*pfkA*, Δ*pfkB*)	*E. coli* JM109 (DE3) harboring pETDuet-*ptsG*-*F*-*mak* and pRSFDuet-*alsE*-*a6PP* with deletion of *fruA*, *pfkA*, and *pfkB*	This study
*E. coli* (Δ*fruA*, Δ*pfkA*, Δ*pfkB*)	*E. coli* JM109 (DE3) harboring pRSFDuet-1 with deletion of *fruA*, *pfkA*, and *pfkB*	This study
*E. coli* (*pckA*, Δ*fruA*, Δ*pfkA*, Δ*pfkB*)	*E. coli* JM109 (DE3) harboring pRSFDuet-*pckA* with deletion of *fruA*, *pfkA*, and *pfkB*	This study
*E. coli* (*alsE*, *a6PP*, *ptsG*-*F*, *mak*, *pckA*, Δ*fruA*, Δ*pfkA*, Δ*pfkB*)	*E. coli* JM109 (DE3) harboring pETDuet-*ptsG*-*F*-*mak* and pRSFDuet-*alsE*-*a6PP*-*pckA* with deletion of *fruA*, *pfkA*, and *pfkB*	This study
Plasmids		
pRSFDuet-1	Vector for protein expression under control of T7 promoter, kan^R^	Novagen
pETDuet-1	Vector for protein expression under control of T7 promoter, Amp^R^	Novagen
pRSFDuet-*a6PP*	pRSFDuet-1 carrying *a6PP*	This study
pRSFDuet-*alsE*	pRSFDuet-1 carrying *alsE*	This study
pRSFDuet-*pckA*	pRSFDuet-1 carrying *pckA*	This study
pRSFDuet-*alsE*-*a6PP*	pRSFDuet-1 carrying *alsE* and *a6PP*	This study
pETDuet-*ptsG*-*F*-*mak*	pETDuet-1 carrying *ptsG*-*F*, and *mak*	This study
pRSFDuet-*alsE*-*a6PP*-*pckA*	pRSFDuet-1 carrying *alsE*, *a6PP and pckA*	This study
pKD46, pKD13, pCP20	λ red recombination system	Guo et al. (2021)

### Plasmid Construction

The plasmids of pETDuet-1 (ampicillin-resistant) and pRSFDuet-1 (kanamycin-resistant) were purchased from Novagen and used to express proteins with T7 promoter. The genes of *ptsG-F* (Kornberg et al., 2000), *mak* (Gene ID 949086), *alsE* (Gene ID 948595), *a6PP* (Gene ID 66330010), and *pckA* (Singh et al., 2011) were synthesized with codon optimization by Sangon Biotech (Shanghai). Cloning of *ptsG*-*F* and *mak* into pETDuet-1 was carried out by use of primers *ptsG*-*F*-F and *ptsG*-*F*-R, *mak*-F and *mak*-R, respectively. The primers for cloning *alsE, a6PP,* and *pckA* into pRSFDuet-1 were *alsE*-F and *alsE*-R, *a6PP*-F and *a6PP*-R, *pckA*-F and *pckA*-R. The primers used in this study were summarized in Table 2. The PCR products were digested with restriction endonucleases (New England Biolabs) and then ligated into plasmids by T4 DNA ligase (New England Biolabs). The recombinant plasmids constructed in this study were shown in Table 1.

**TABLE 2:** The primers used in this study.

Name	Primer sequence (5′→3′)	Description
Plasmid construction		
*ptsG*-*F*-F	CGGGA​TCCGAT​GTT​TAA​GAA​TGC​ATT​TGC​TAA​C	Clone and insert *ptsG*-*F* into *BamH* I and *Hind* III on pETDuet-1
*ptsG*-*F*-R	CCCAAG​CTTTTA​GTG​GTT​ACG​GAT​GTA​CTC	
*mak*-F	GGA​ATT​CCAT​ATGGTG​CGT​ATA​GGT​ATC​G	Clone and insert *mak* into *Nde* I and *Kpn* I on pETDuet-*ptsG*-*F*
*mak*-R	GGGGT​ACCTTA​CTC​TTG​TGG​CCA​TAA​CCA​CGC	
*a6PP*-F	GGA​ATT​CCAT​ATGAAA​TAC​ACC​GTT​TAC​CTG​TTC​G	Clone and insert *a6PP* into *Nde* I and *Kpn* I on pRSFDuet-1
*a6PP*-R	GGGGT​ACCTTA​CAG​CGG​GCA​ACC​AGA​TTT​ATC​TTC	
*alsE*-F	CAT​GCCA​TGGGCA​TGA​AAA​TCT​CCC​CCT​C	Clone and insert *alsE* into *Nco* I and *Hind* III on pRSFDuet-1 or pRSFDuet-*a6PP*.
*alsE*-R	CCCAAG​CTTTTA​TGC​TGT​TTT​TGC​ATG​AG	
*pckA*-F	CCGCTC​GAGCCT​GTA​GAA​ATA​ATT​TTG​TTT​AAC​TTT​AAT​AAG​GAG​ATA​TAC​CAT​GAG​CTT​ATC​TGA​AAG	Clone and insert *pckA* into *Xho* I and *Avr* II on pRSFDuet-1 or pRSFDuet-*a6PP*-*alsE*
*pckA*-R	CCGCCT​AGGTTA​TAA​CTG​TGG​ACC​AGC​C	
Gene deletion		
*fruA*-F	CTG​ACA​GCA​GGA​GAG​GCA​TAA​TGA​AAA​CGC​TGC​TGA​TTA​TTG​ACG​CTA​ATA​TTC​CGG​GGA​TCC​GTC​GAC​C	Delete *fruA*
*fruA*-R	GCC​CTG​TAA​CAC​ACC​TTT​TAT​TAC​GCT​GCT​TTC​GCT​ACT​GCG​TCC​ACT​TCG​TGT​AGG​CTG​GAG​CTG​CTT​CG	
*pfkA*-F	GTT​CAG​AGG​TAG​TCA​TGA​TTA​AGA​AAA​TCG​GTG​TGT​TGA​CAA​GCG​GCG​GTT​GTG​TAG​GCT​GGA​GCT​GCT​TCG	Delete *pfkA*
*pfkA*-R	CGA​AAT​CAT​TAA​TAC​AGT​TTT​TTC​GCG​CAG​TCC​AGC​CAG​TCA​CCT​TTG​AAA​TTC​CGG​GGA​TCC​GTC​GAC​C	
*pfkB*-F	CTG​ATT​CGG​TGC​CAG​ACT​GAA​ATC​AGC​CTA​TAG​GAG​GAA​ATG​ATG​GTA​CGT​ATC​TGT​GTA​GGC​TGG​AGC​TGC​TTC​G	Delete *pfkB*
*pfkB*-R	GTT​GGT​GAT​GAT​TCC​CCC​AAT​GCT​GGG​GGA​ATG​TTT​TTG​TTA​GCG​GGA​AAG​GAT​TCC​GGG​GAT​CCG​TCG​ACC	

### Protein Expression and Enzymatic Analysis

*E. coli* cells were grown in 200 ml LB medium with kanamycin (50 μg/ml) at 37°C and 220 rpm. After 3 h of cultivation, we added 0.4 mM isopropyl-β-D-thiogalactoside (IPTG) to the medium to induce the expression of A6PP or AlsE for 12 h. Cells were harvested by centrifugation at 4°C and 6,000 *g* for 10 min and re-suspended in Tris-HCl buffer (pH 7.5, 50 mM), which were then broken by use of an ultrasonic cell crusher JY92-IIN (Jingxin, Shanghai). The sonication was performed on ice for 5 min (2 s pulse on and 2 s pulse off) at 50% amplitude. Cell debris was removed by centrifugation at 4°C and 6,000 *g* for 5 min. Sodium dodecyl sulfate–polyacrylamide gel electrophoresis (SDS–PAGE) was carried out using 8% polyacrylamide gels (Jinruisi Biotech, Nanjing) for analyzing the supernatant. We added the crude enzyme (20 μl, 10 mg/ml) to 1 ml Tris-HCl buffer (pH 7.5, 50 mM) containing 2.60 g/L fructose-6-phosphate, and the reaction was conducted at 37°C for 30 min for enzyme assay.

### Gene Knockout

Deletion of *fruA* (Gene ID 946672), *pfkA* (Gene ID 948412), and *pfkB* (Gene ID 946230) was achieved by a *λ* red homologous recombination system (Datsenko and Wanner, 2000). The DNA fragments were amplified with pKD13 (kanamycin-resistant) as a template (Baba et al., 2006), and the primers were listed in Table 2. *E. coli* JM109 (DE3) harboring pKD46 (ampicillin-resistant) were grown in LB medium with ampicillin (100 μg/ml) at 30°C and 220 rpm, until the cell density (OD_600_) reached ≈0.3. Then L-arabinose (4 g/L) was added to induce the expression of homologous recombinase at 37°C and 220 rpm for 1 h (Yang et al., 2018). The DNA fragments were electroporated into the L-arabinose-induced cells for gene knockout. The plasmid of pCP20 (ampicillin and chloramphenicol-resistant) was used to remove the kanamycin resistance gene in recombinant cells (Dugar et al., 2016).

### Fermentation

*E. coli* cells were inoculated in 4 ml LB medium containing appropriate antibiotics and grown overnight at 37°C and 220 rpm. Cells were transferred to the flask with 50 ml LB medium containing appropriate antibiotics and 4.00 g/L D-fructose. Potassium phosphate (100 mM) was used to maintain the pH of the fermentation broth, and IPTG (0.4 mM) was added to induce protein expression. Air-limited fermentation was carried out in the airtight bottle with either buffered-LB medium or M9 minimal medium containing appropriate antibiotics, 2.20 g/L D-fructose, 8.00 g/L glycerol, and 0.4 mM IPTG.

### Analytical Approach

Cell density was determined by a microplate reader (Multiskan™ FC, Thermo Scientific). The ATP in cells was measured through an ATP Content Assay Kit (BC0300, Solarbio) as previously reported (Wang et al., 2019). D-Allulose, D-fructose, and glycerol were determined using a high-performance liquid chromatograph (HPLC, HITACHI) equipped with a refractive index detector (RID) monitor. Sugar-Pak™ I Column (85°C, Waters) was used with deionized water as the mobile phase at a flow rate of 0.5 ml/min.

## Results and Discussion

### Biosynthesis of D-Allulose Through Phosphorylation-Dephosphorylation

Wild-type *E. coli* is able to grow on D-fructose as a carbon source owing to its fructose PTS and mannose PTS (Kornberg, 2001). The D-fructose passaged *via* mannose PTS appears in cells as fructose-6-phosphate (Luo et al., 2014), which can be epimerized to allulose-6-phosphate due to the presence of allulose-6-phosphate epimerase (*alsE*) (Chan et al., 2008). Therefore, the key to establishing a phosphorylation-dephosphorylation pathway in *E. coli* for D-allulose synthesis is the introduction of an appropriate allulose-6-phosphate phosphatase for allulose-6-phosphate dephosphorylation.

**FIGURE 2:**
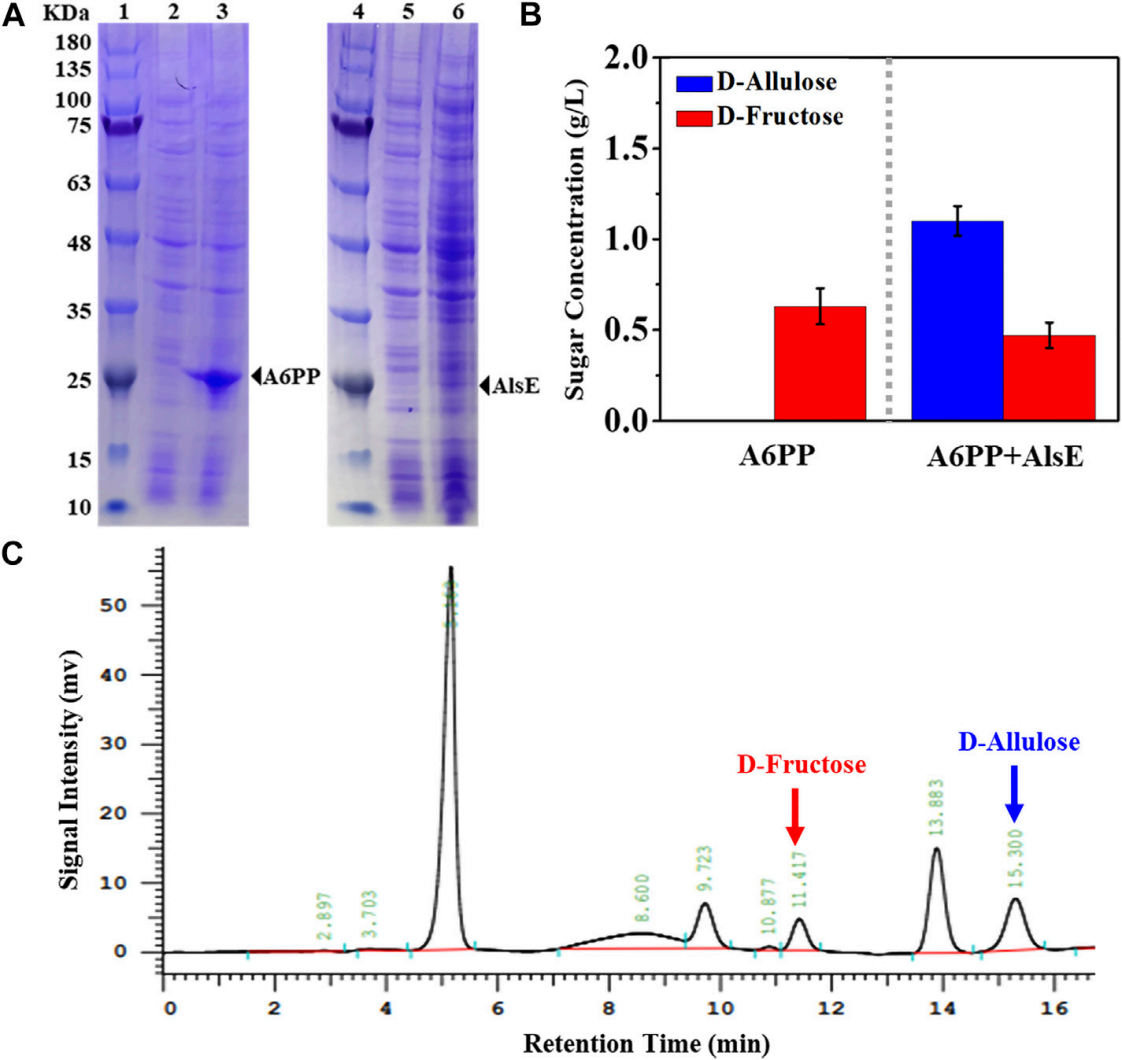
Expression and enzyme assay of A6PP and AlsE. **(A)** SDS-PAGE analysis. Marker (lane 1 and 4), *E. coli* (*control*) (lane 2 and 5), *E. coli* (*a6PP*) (lane 3), *E. coli* (*alsE*) (lane 6). **(B)** Conversion of D-fructose to D-allulose using crude A6PP or a mixture of crude A6PP and AlsE. The reaction was carried out in Tris-HCl buffer (pH 7.5, 50 mM) containing 2.60 g/L fructose-6-phosphate at 37°C for 30 min. Error bars, *SD*, *n = 3*. **(C)** HPLC analysis for confirming the generation of D-allulose by use of A6PP and AlsE mixture.

**FIGURE 3:**
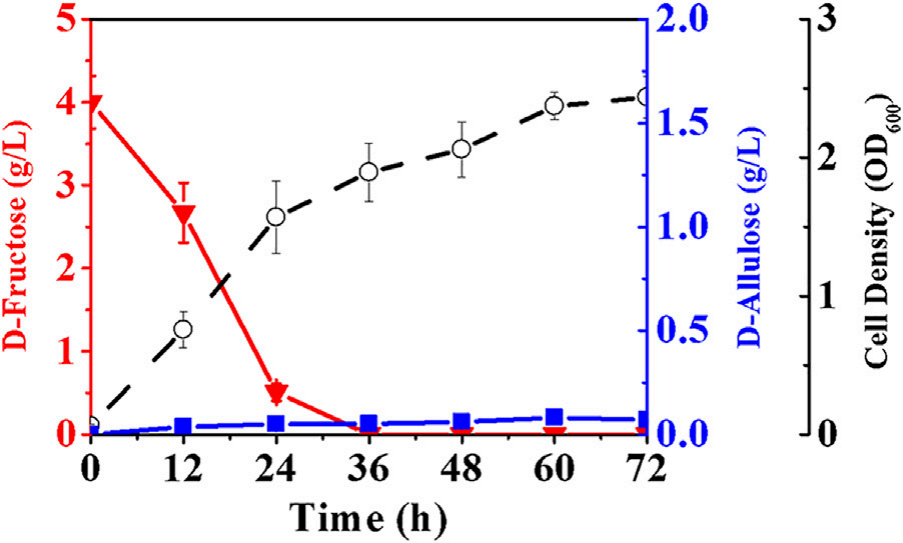
Production of D-allulose by *E. coli* co-expressing AlsE and A6PP. *E. coli* (*alsE*, *a6PP*) was cultured in LB medium with 4.00 g/L D-fructose at 37°C. Error bars, *SD*, *n = 3*.

We selected an allulose-6-phosphate phosphatase (*a6PP*) from *B. fragilis* since it exhibited a high activity at moderate temperatures. Expression of *a6PP* was carried out in *E. coli* JM109 (DE3), resulting in the strain *E. coli* (*a6PP*). As shown in Figure 2A, the SDS-PAGE results indicate that this enzyme was expressed in a soluble form with a molecular mass of ≈25 kDa. Unfortunately, it was found that *E. coli* (*a6PP*) could not produce D-allulose by fermentation even if D-fructose was supplemented in culture medium (LB), which is in good agreement with the experimental results of the *in vitro* assay (Figure 2B). We sonicated *E. coli* (*a6PP*) after induction and utilized the supernatant to catalyze the cascade of epimerization and dephosphorylation using fructose-6-phosphate as a substrate, and observed the absence of target product in the reaction system. Although we reconfirmed that the genome of *E. coli* JM109 (DE3) has the gene of *alsE*, its weak expression in cells might be unable to generate sufficient allulose-6-phosphate from fructose-6-phosphate. Therefore, we cloned and over-expressed *alsE* by use of *E. coli* (*alsE*), finding the molecular mass of AlsE was close to that of A6PP, but its expression level was quite low under the same promoter and induction conditions (Figure 2A). When a mixed crude enzyme solution of AlsE and A6PP was tested *in vitro*, it could produce 1.10 g/L D-allulose and 0.47 g/L D-fructose from 2.60 g/L fructose-6-phosphate within 30 min (Figures 2B,C), which demonstrates the functionalities of AlsE and A6PP in the pathway from fructose-6-phosphate to D-allulose and confirms the presence of an activity of A6PP on fructose-6-phosphate dephosphorylation. Then we co-expressed *alsE* and *a6PP* in *E. coli* JM109 (DE3), resulting in the strain *E. coli* (*alsE*, *a6PP*). Figure 3 shows that 0.07 g/L D-allulose could be generated when *E. coli* (*alsE*, *a6PP*) was cultured in the LB medium supplemented with 4.00 g/L D-fructose at 37°C for 60 h.

### Regulation of Metabolic Pathways to Increase Cell Factory Efficiency

**FIGURE 4:**
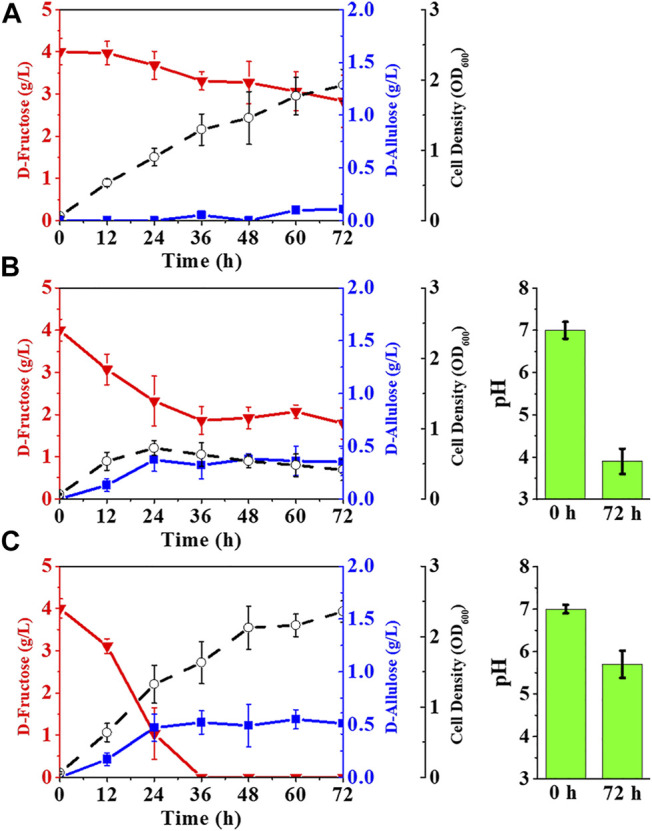
Reprogramming of fructose transport pathways in *E. coli* to enhance D-allulose production. *E. coli* cells were cultured in LB medium with 4.00 g/L D-fructose at 37°C. **(A)** *E. coli* (*alsE*, *a6PP*, Δ*fruA*), **(B)** *E. coli* (*alsE*, *a6PP*, *ptsG*-*F*, *mak*, Δ*fruA*), **(C)** *E. coli* (*alsE*, *a6PP*, *ptsG*-*F*, *mak*, Δ*fruA*) with 100 mM potassium phosphate. Error bars, *SD*, *n = 3*.

Although *E. coli* (*alsE*, *a6PP*) was capable of producing D-allulose from D-fructose by fermentation, the titer and yield were quite low. The fructose PTS has been reported to play a predominant role in D-fructose transport and phosphorylation in *E. coli* (Luo et al., 2014), so most of the D-fructose entering cells should be in the form of fructose-1-phosphate rather than fructose-6-phosphate. Fructose-1-phosphate is not a biosynthetic precursor for the D-allulose synthesis pathway and can only flow into the EMP pathway as a carbon source for cell growth. We thus deleted the gene of *fruA* to damage the fructose PTS in *E. coli* (*alsE*, *a6PP*). As illustrated in Figure 4A, the fermentation performance of *E. coli* (*alsE*, *a6PP*, Δ*fruA*) was slightly improved, with a D-allulose titer of 0.11 g/L and a yield of 0.09 g/g. However, D-fructose could not be depleted, and over 71% remained in the LB medium after 72 h. It is probable that the loss of fructose PTS severely reduced the ability of cells to take up D-fructose. We then employed the gene of *ptsG*-*F* to construct a facilitated diffusion passageway for D-fructose transport and the gene of *mak* for subsequent phosphorylation at its C-6 position (Kornberg et al., 2000). The D-allulose titer of *E. coli* (*alsE*, *a6PP*, *ptsG*-*F*, *mak*, Δ*fruA*) reached 0.35 g/L, with a product yield of 0.16 g/g (Figure 4B). In this case, we found an obvious deterioration in cell growth, probably due to the sharp drop in pH during fermentation, which was caused by the dephosphorylation of fructose-6-phosphate. As shown in Figure 4C, buffering LB medium with 100 mM potassium phosphate was able to limit this acidification to pH 5.7 after 72 h, and the D-allulose titer increased to 0.51 g/L, with depletion of 4.03 g/L D-fructose.

**FIGURE 5:**
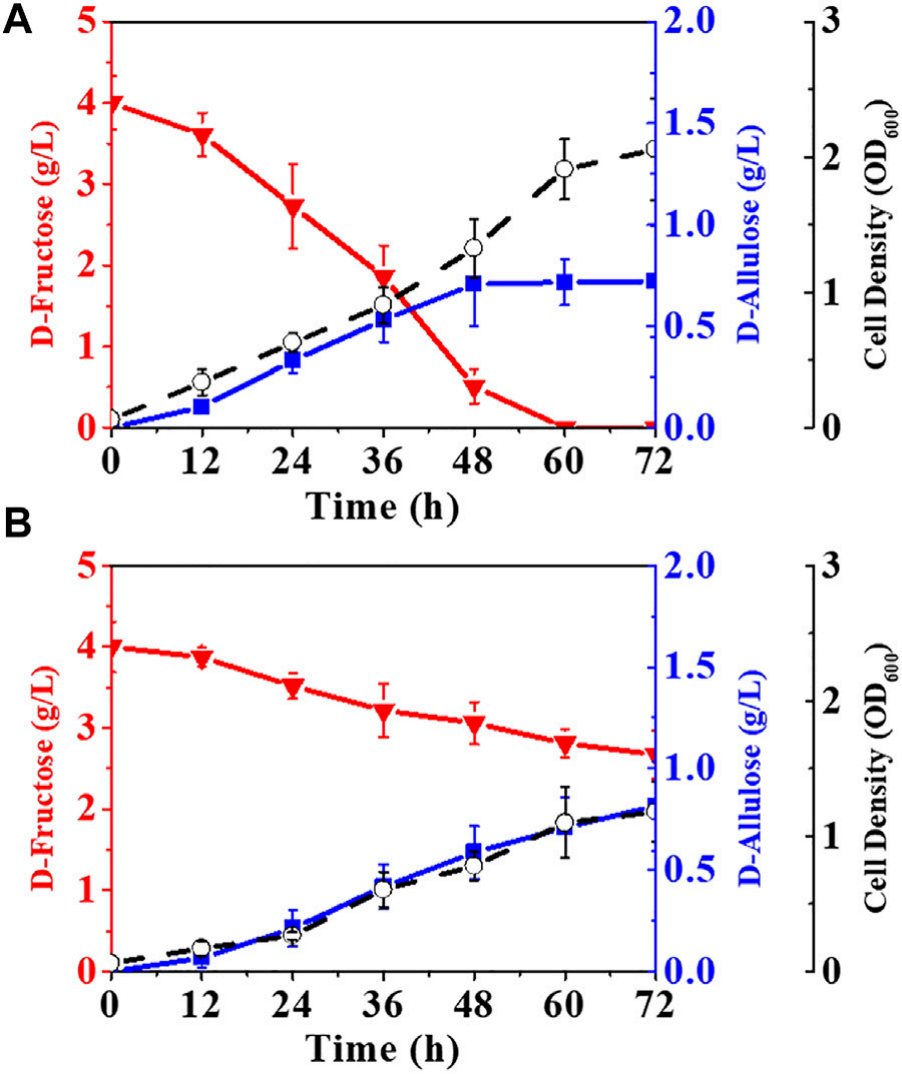
Elimination of the pathways phosphorylating fructose-6-phosphate. *E. coli* cells were cultured in LB medium with 4.00 g/L D-fructose and 100 mM potassium phosphate at 37°C. **(A)** *E. coli* (*alsE*, *a6PP*, *ptsG*-*F*, *mak*, Δ*fruA*, Δ*pfkA*), **(B)** *E. coli* (*alsE*, *a6PP*, *ptsG*-*F*, *mak*, Δ*fruA*, Δ*pfkA*, Δ*pfkB*). Error bars, *SD*, *n = 3*.

In *E. coli* cells, fructose-6-phosphate can also be phosphorylated into fructose-1, 6-bisphosphate by 6-phosphofructokinases (*pfkA*, *pfkB*) and utilized as a carbon source through the EMP pathway (Wang et al., 2022). Therefore, blocking the phosphorylation of fructose-6-phosphate may favor the synthesis of D-allulose. The results in Figure 5A show that deletion of *pfkA* in *E. coli* (*alsE*, *a6PP*, *ptsG*-*F*, *mak*, Δ*fruA*) increased the D-allulose titer and yield to 0.72 g/L and 0.18 g/g, respectively. When both *pfkA* and *pfkB* were knocked out (Figure 5B), the titer and yield were further improved, especially the product yield could reach 0.61 g/g on D-fructose, but it significantly affected cell growth, probably due to the limitation of the carbon flux into the EMP pathway. Moreover, the balance of the endogenous gene expression in *E. coli* cells might be affected after gene knockout, and a reported solution was to construct gene-inactivated libraries (Du et al., 2021; Zhu et al., 2021).

### Coupling of ATP Regeneration System to Improve Cofactor Supply

Replacing the fructose PTS with *ptsG*-*F* and *mak* allowed D-fructose to be transported and concomitantly phosphorylated to fructose-6-phosphate, a precursor for D-allulose synthesis. Another consequence of this substitution was that the phosphate donor required for D-fructose phosphorylation changed from PEP to ATP, so the D-allulose titer of *E. coli* should be closely tied to the ability of cell factory to provide ATP. Our results show that deletion of *pfkA* and *pfkB* increased the product yield by blocking the entry of fructose-6-phosphate into the EMP pathway, but it might lead to a restriction in cellular ATP generation. To maximize D-allulose level, it is essential to develop a complementary strategy that yields ATP.

**FIGURE 6:**
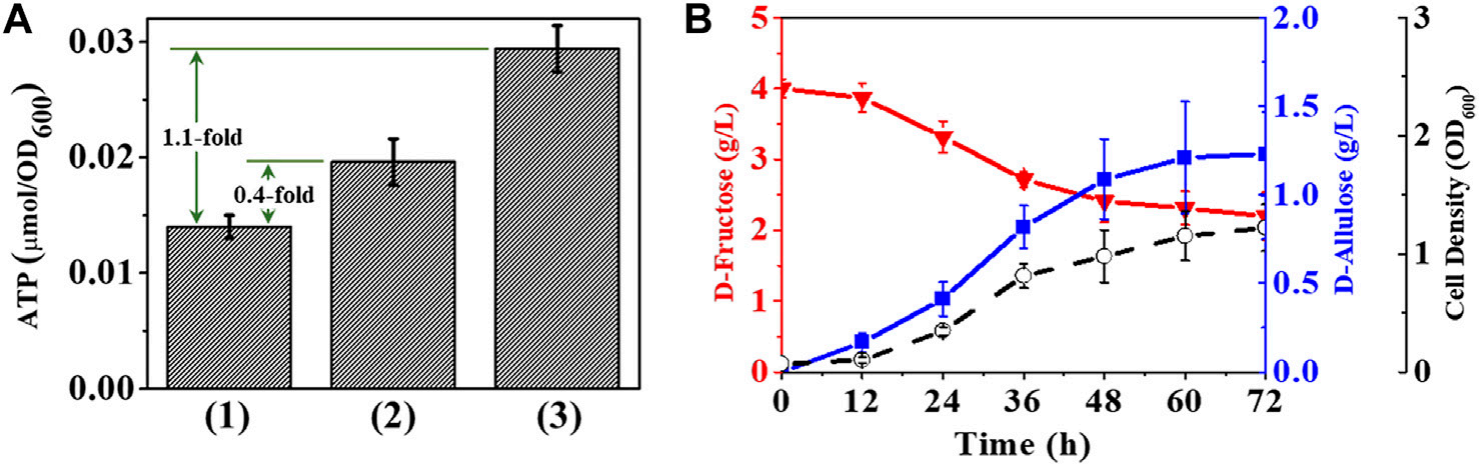
Improvement of ATP supply in cell factory. **(A)** Intracellular ATP level, 1) *E. coli* (Δ*fruA*, Δ*pfkA*, Δ*pfkB*), 2) *E. coli* (*pckA*, Δ*fruA*, Δ*pfkA*, Δ*pfkB*), 3) *E. coli* (*pckA*, Δ*fruA*, Δ*pfkA*, Δ*pfkB*) under air-limited condition. **(B)** *E. coli* (*alsE*, *a6PP*, *ptsG*-*F*, *mak*, *pckA*, Δ*fruA*, Δ*pfkA*, Δ*pfkB*) cultured in buffered-LB medium with air-limitation condition at 37°C. Error bars, *SD*, *n = 3*.

Since inactivation of fructose PTS was believed to increase the PEP pool in *E. coli* cells (Luo et al., 2014), it should be a prior consideration to utilize PEP-related reactions to enhance ATP formation. OAA is a source of four-carbon dicarboxylic acid for the tricarboxylic acid (TCA) cycle and can be formed reversibly from PEP in *E. coli* by using PEP-carboxylase (*ppc*) (Chatterjee et al., 2001; Lin et al., 2005). In contrast, the conversion of PEP to OAA in *A. succinogenes* is catalyzed by PEP-carboxykinase (*pckA*) (Singh et al., 2011), which requires ADP as a phosphate acceptor and thus facilitates ATP generation in cells. We then expressed *pckA via* T7 promoter in *E. coli* (Δ*fruA*, Δ*pfkA*, Δ*pfkB*) and measured the changes in cellular ATP content. The data in Figure 6A show that use of *pckA* resulted in a 0.4-fold increase in ATP level, whereas this increase was able to rise to 1.1-fold by reducing air supply during cell cultivation, which is consistent with previous findings that PEP-carboxykinase catalyzed the reversible reaction towards the ATP-generating direction at high CO_2_ concentrations (Singh et al., 2011). Figure 6B illustrates that the D-allulose titer and yield of *E. coli* (*alsE*, *a6PP*, *ptsG*-*F*, *mak*, *pckA*, Δ*fruA*, Δ*pfkA*, Δ*pfkB*) grown in buffered-LB medium with air-limitation condition reached 1.23 g/L and 0.68 g/g, respectively, as a result of increased ATP inside cells.

### Production of D-Allulose in Minimal Medium With Air-Limited Condition

After achieving the construction and optimization of the cell factory pathways, we hoped to further improve the production of D-allulose through fermentation. The major problem encountered was cell growth defect caused by the block of the EMP pathway. Although LB is a nutritionally-rich medium in which yeast extract can act as a carbon source for *E. coli*, the developed cell factory did not grow well, with a cell density of less than 1.22 after 72 h (Figure 6B). Also, this medium has a relatively low buffering capacity that is not conducive to the cells engineered with dephosphorylation pathway, and is normally used to produce enzymes rather than synthetic chemicals due to its high cost. M9 minimal medium may be a better choice compared to LB, but the premise of using M9 in our case is to select a suitable carbon source that can be utilized by *E. coli* cells without the participation of 6-phosphofructokinases.

**FIGURE 7:**
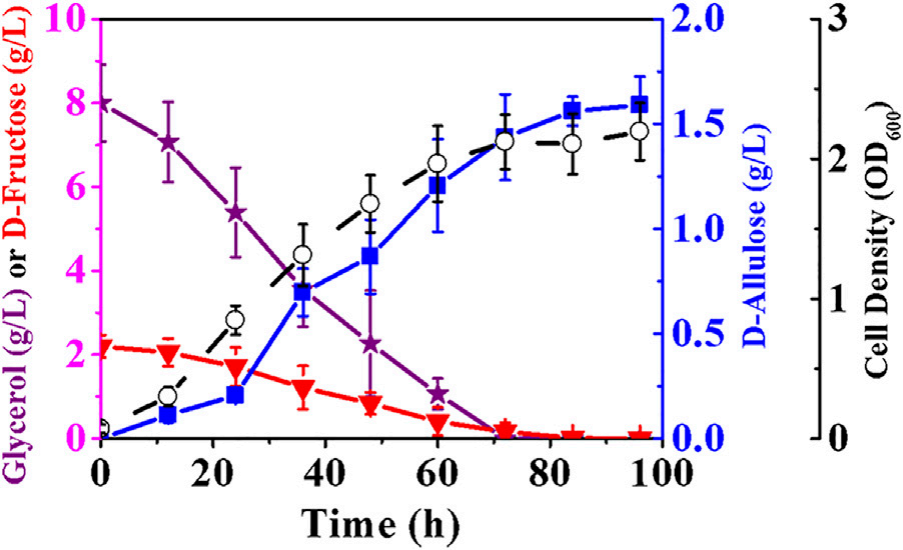
Production of D-allulose in minimal medium with air-limited condition. *E. coli* (*alsE*, *a6PP*, *ptsG*-*F*, *mak*, *pckA*, Δ*fruA*, Δ*pfkA*, Δ*pfkB*) was cultured in M9 minimal medium with 2.20 g/L D-fructose and 8.00 g/L glycerol at 37°C. Error bars, *SD*, *n = 3*.

Theoretically, *E. coli* (*alsE*, *a6PP*, *ptsG*-*F*, *mak*, *pckA*, Δ*fruA*, Δ*pfkA*, Δ*pfkB*) should restore its growth on glycerol, an abundant three-carbon by-product of biodiesel industry. Glycerol can be metabolized to dihydroxyacetone phosphate (DHAP) via either fermentative or respiratory route, and the conversion of DHAP to PEP requires only the EMP pathway downstream of 6-phosphofructokinases (Chiang et al., 2020; Zhan et al., 2020). Therefore, we attempted to produce D-allulose with glycerol-containing M9 medium and air-limited condition. In order to fully exhibit the advantages of phosphorylation-dephosphorylation over Izumoring in substrate conversion ratio, the initial D-fructose concentration in medium was controlled at 2.20 g/L. The data in Figure 7 show that glycerol was utilized by *E. coli* (*alsE*, *a6PP*, *ptsG*-*F*, *mak*, *pckA*, Δ*fruA*, Δ*pfkA*, Δ*pfkB*) as expected, and cell growth defect was not observed, resulting in a cell density of 2.21 after 100 h. The favorable growth conditions increased the titer of D-allulose to 1.59 g/L. It should be noted that D-fructose could be exhausted after fermentation, and most of it was used for synthesis of the target product, with a yield of 0.72 g/g.

Our achievements in this study perfectly overcome the D-fructose conversion bottleneck existing in fermentative production of D-allulose, and suggest a breakthrough in application of phosphorylation-dephosphorylation strategy in cell factories. Currently, the conversion ratio of D-fructose using Izumoring-based cell factories was only 19.6% (Guo et al., 2021). In contrast, the new synthetic route can completely consume D-fructose, which may facilitate the subsequent separation of D-allulose from culture broth. In future work, we plan to optimize the expression of allulose-6-phosphate epimerase by gene screening or tag fusion, which may greatly increase D-allulose titer while maintaining a high product yield.

## Data Availability

The original contributions presented in the study are included in the article/supplementary material, further inquiries can be directed to the corresponding authors.
